# The Characteristics of Canadian University Students’ Mental Health, Engagement in Activities and Use of Smartphones: A descriptive pilot study

**DOI:** 10.1177/20551029211062029

**Published:** 2021-12-13

**Authors:** Behdin Nowrouzi-Kia, Jill Stier, Luma Ayyoub, Lauren Hutchinson, Jamie Laframboise, Alex Mihailidis

**Affiliations:** 1 Department of Occupational Science and Occupational Therapy, 12366 Temerty Faculty of Medicine, University of Toronto, Toronto, ON, Canada; 2 Rehabilitation Sciences Institute, 12366 Temerty Faculty of Medicine, University of Toronto, Toronto, ON, Canada; 3 Toronto Rehab, University Health Network, Toronto, ON, Canada

**Keywords:** Mental health/psychiatry, university students, smartphones, information and communication technology, Canada

## Abstract

**Background:**

Mental health issues are on the rise which may impede university students’ abilities to perform daily functions and interact with other community members. The objectives of the current study are to explore (1) the characteristics of university students’ mental health and engagement in activities, (2) how students use their smartphones to support their mental health and engagement in activities, (3) student preferences for important features and functions of a smartphone application (app) that promote engagement in activities and (4) student perspectives about what data an app should collect as indicators of change in their mental health and engagement in activities.

**Methods:**

We designed a pilot study and an online questionnaire with open and closed-ended questions to collect data exploring the association between student mental health and engagement in activities. The questionnaire included four sections: demographics, mental health and activity status and management, general smartphone use, and smartphone use to support mental health and engagement in activities. The data were analysed using descriptive statistics.

**Results:**

A total of 56 participants were recruited to complete the online survey, with an average completion rate of 77% (*n* = 43). The majority of participants were 24 years of age or older (*n* = 34, 65.4%), and less than half were between the ages of 18 and 23 (*n* = 18, 34.6%). The results of participants’ engagement in self-care, productivity and leisure/play activities are reported. As well, participants’ use of smartphones to support their mental health is described.

**Conclusions:**

This study provides a greater understanding of what features and functions to include and what data to collect when developing a novel app to support students’ mental health and engagement in activities. Moreover, it clarifies the bidirectional relationship between mental health changes and self-care engagement, productivity/work and leisure/play domains.

## Background

Mental health issues may impede university students’ abilities to perform daily functions and interact with other community members (Giamos et al., 2017). Current approaches (e.g. self-report) are insufficient and do not meet the rising needs of mental health monitoring and treatment on university campuses (Boukhechba et al., 2018). Several applications (apps) as well as information and communication technology (ICT) based approaches have been developed in response to the rising demand for mental health services. ICT includes a variety of technologies that enable the exchange of data through the internet (e.g. telehealth/telemedicine) or telephone (e.g. telephone triage) (While & Dewsbury, 2011). However, these applications typically focus on providing interventions instead of determining and predicting who may be at risk for mental health issues. Typically, these interventions are missing pertinent information delivered too late and not fully effective.

A study of 58 Canadian post-secondary institutions reported that within the last 12 months, 19.6% felt overwhelmed by all they had to do and 24.6% of respondents felt hopeless (American College Health Association, 2019). In Ontario, university students are twice as likely to report mental illness symptoms than non-university individuals (Ontario College Health Association, 2009). Nearly a quarter (24.6%) reported feeling hopeless, and 21.2% indicated feeling so depressed they were unable to function (American College Health Association, 2019). Furthermore, approximately 80% of Ontario-based students felt overwhelmed or exhausted within the last 12 months (Ontario College Health Association, 2009). However, only 7.4% of the students reported they had been diagnosed or treated for a mental health condition by a healthcare professional during the same period (American College Health Association, 2016). With the increased prevalence of mental health conditions at universities, many existing services cannot meet students’ needs.

ICT solutions may offer potential strategies and solutions in addressing university students’ mental health needs (Gagnon et al., 2012). For example, the *StudentLife* app assessed the day-to-day and week-by-week impact of workload on stress, sleep, activity, mood, sociability, mental well-being and academic performance of 48 students at Dartmouth College using Android phones (Wang et al., 2014). Wang et al. (2014) found significant correlations between objective sensor data from smartphones and mental well-being (e.g. self-reported depression and stress) and academic performance (e.g. number of assignments). Other studies have reported using ICT, such as short message service (SMS), to remind patients of their health appointments (Branson et al., 2013); to foster conversation between patients and healthcare practitioners (Clough & Casey, 2015; Furber et al., 2011; Nolan et al., 2011); or to deliver relaxation interventions to university students (Grassi et al., 2009; Riva et al., 2007). Other studies have supported a link between ICT and new aspects of clinically relevant conditions like depression (Insel, 2017) and schizophrenia (Barnett et al., 2018). However, these and other previously developed devices do not predict mental health changes, as proposed in this new study.

Common features of existing mental health apps include the provision of specific diagnoses such as depression and anxiety (Chawathey & Ford, 2016; Donker et al., 2013; Grassi et al., 2009; Watts et al., 2013) and do not focus on the relationship between mental health changes and engagement in activities (Bar & Jarus, 2015). Additionally, these apps do not fully use the smartphone sensory capabilities that could be used to track changes in students’ mental health.(Wang et al., 2014) Finally, during app development, user perspectives on vital features and functions are not considered which negatively affects user agreeability and satisfaction with products (McCurdie et al., 2012). For example, when users are engaged throughout the app developmental cycle, several essential system requirements may be elucidated and therefore, a user-centred design processes in the use of mobile technologies to support health objectives (McCurdie et al., 2012) Moreover, findings from McCurdie et al. (2012) outline the need to understand user needs and how data collected by a smartphone can be used to develop a novel app that can alert post-secondary students to changes in their mental health so they can be best supported.

The objectives of the current study are to explore (1) the characteristics of student mental health and engagement in activities, (2) how students use their smartphones to support their mental health and engagement in activities, (3) student preferences for important features and functions of a smartphone application (app) that supports their mental health and engagement in activities, and (4) student perspectives about what data an app should collect as indicators of change in their mental health and engagement in activities.

## Methods

### Design

We designed a pilot study and an online questionnaire with both open and closed-ended questions to collect data that explored the association between student mental health and engagement in activities. Ethics approval for human research was obtained from the Institutional review board at the University of Toronto. All participants provided informed electronic written consent.

### Sampling frame

Participants recruited were students at a Canadian university who were 18 years and older and enrolled in undergraduate or graduate studies on a full-time or part-time basis. A chain sampling approach was used to recruit participants (e.g. asking one participant if they know of someone else who would be a suitable participant in the study). Participants were not asked to provide any identifying information on the online questionnaire so that responses remained anonymous. Data obtained from the questionnaire for data analysis were extracted from SurveyMonkey by one of the co-authors (AM) and stored securely at the University’ secure server. The medical literature suggests a sample size of 12 participants (Connelly, 2008; Julious, 2005) or approximately 10% of the project’s sample size (Connelly, 2008).

### Data collection

Data were collected using an online questionnaire to understand what end users believed were important features and functions of an app that predicts student mental and engagement changes. The questionnaire included four sections: demographics, mental health and activity status and management, general smartphone use, and smartphone use to support mental health and engagement in activities. Participants were contacted through social media advertisements with no prenotifications or reminders. Participants were not provided with an incentive.

Since this questionnaire was administered during the coronavirus disease 2019 (COVID-19) pandemic, participants were asked to answer questions about their mental health by referring to the last 6 months before the COVID-19 quarantine. The demographics section included participants’ age, gender, year of study, enrolment type (full-time or part-time), which campus of the university they were enrolled in, employment, and living situation. The mental health status and management section included general questions about mental health status, for example, ‘in the last 6 months, how often did you feel stressed, anxious, sad, or depressed?’ where participants rated their answer on a 5-point Likert scale (never, rarely, sometimes, often and always); and ‘have you ever used your smartphone to support your mental health?’.

The mental health and activity status and management section included three questions modified from the World Health Organization – Disability Assessment Schedule 2.0 (WHO-DAS 2.0) (World Health Organization, 2020) which were related to the impact of the participants’ mental health on their functional activities domains. Participants were asked about three main areas of activities-self-care, productivity/work, and leisure/play – each of which had specific occupations to rate.

The third section of the survey asked general questions about smartphone use. Participants were surveyed on their use of different smartphone applications and tools in the areas of activity (self-care, productivity/work and leisure/play). These categories were based on the belief that all activities are divisible into three categories: 1) self-care (e.g. dressing, eating, bathing, sleeping, grooming), 2) productivity/work (e.g. going to work or school) and 3) leisure/play (e.g. social activities, physical activity, hobbies).(Canadian Association of Occupational Therapy, 2016; Kielhofner, 2002; Townsend & Polatajko, 2007) For example, participants were asked, ‘Which of the following smartphone tools do you use to: socialize, for work or school purposes; to manage your finances, and to manage your health and fitness?’. Finally, the last section of the survey asked participants about their use of smartphone apps and tools to support their mental health and what data would be useful to collect to support mental health. For example, participants were asked, ‘Have you ever used your smartphone to support your mental health?’, and ‘Do you use apps to support your mental health?’. The questionnaire also asked about student preferences of smartphone apps and tools that support mental health. Students were asked to rate the importance of different features included in an app. These features included cost, ease of use, aesthetics of application interface, ability to set-up features and customize operation, and the ability for the system to work without manual input from the user. Other questions were also asked in this section to gain an understanding of what students perceive are predictors of changes in their mental health and engagement in an activity.

### Data analysis

The data was analysed in R software 4.02 for Windows.(Team, 2015; Viechtbauer, 2020) Descriptive statistics, including means and proportions, were used to characterize the respondents by demographic variables, mental health and activity status and management, general smartphone use, and smartphone use to support mental health and engagement in activities. The completion rate of the study was determined by the number of completed surveys. Missing data was coded and identified in the dataset using predefined variables.

## Results

### Characteristics of participants

A total of 56 participants were recruited to complete the online survey, with an average completion rate of 77% (*n* = 43). The majority of participants were 24 years of age or older (*n* = 34, 65.4%), and less than half were between the ages of 18 and 23 (*n* = 18, 34.6%). Ninety-two percent of respondents were female students (*n* = 48), and 66.1% were students at the graduate level (*n* = 37). Of the participants, 64.7% (*n* = 33) reported being unemployed in the last 6 months, and 35.3% (*n* = 1) reported being employed as either part-time (*n* = 17) or full-time (*n* = 1). 13 percent (13.7%) of participants (*n* = 7) reported living alone while attending their studies. The majority of students reported feeling stressed, anxious, sad, or depressed sometimes (*n* = 23, 48.9%) or very often (*n* = 22, 46.8%) in the last six months, and 36.2% (*n* = 17) of participants self-identified as someone with a mental health condition (Table 1).

**Table 1.** Characteristics of participants.

Variable	(*n* = 57)	
	*n* ^a^	%
Age		
18–20	2	3.0
20–22	0	0
22–24	16	30.8
24–26	25	48.1
>26	9	17.3
Year of university study		
Year 1 of undergraduate degree	4	8
Year 2 of undergraduate degree	9	18.0
Year 3 of undergraduate degree	0	0
Year 4 of undergraduate degree	0	30.9
Year 5 of undergraduate degree	0	68.5
Graduate studies	37	74.0
Gender		
Female	48	92.3
Male	3	5.8
Undeclared	1	0.0
Other	0	1.9
Employment status		
Full-time (30 h or more per week)	1	2.0
Part-time (less than 30 h per week)	17	33.3
Unemployed	33	64.7
Who do you live with while attending your studies		
Family	22	43.1
Spouse or partner	5	9.8
Roommate(s)	16	31.4
Alone	7	13.7
Other	1	2.0
Do you self-identify as someone with a mental health condition		
Yes	17	36.2
No	39	59.6
Prefer not to answer	2	4.3
In the past 6 months, how often did you feel stressed, anxious, sad or depressed?		
Always	1	2.1
Very often	22	46.8
Sometimes	23	48.9
Rarely	1	2.1
Never	0	0
Prefer not to answer	0	0

^a^Totals for each variable do not add to 57 because missing values were excluded from calculations.

### Student engagement in functional activities

#### Self-care

A total of 47 participants reported how stress, anxiety, sadness or depression were to impact their engagement in self-care activities in the last 6 months. Participants reported that stress, anxiety, sadness or depression ‘never’ (*n* = 62, 26.4%), ‘rarely’ (*n* = 65, 27.7%), ‘sometimes’ (*n* = 75, 31.9%), ‘often’ (*n* = 30, 12.8%) or ‘always’ (*n* = 3, 1.3%) impacted their engagement in self-care activities (Table 2). Participants rated that their feelings would ‘sometimes’ (*n* = 19, 40.43%) or ‘often’ (*n* = 15, 31.91%) impact their engagement in sleeping, and ‘sometimes’ (*n* = 23, 48.94%) or ‘often’ (*n* = 8, 17.02%) impact their engagement with eating.

**Table 2.** Student engagement in functional activities (*n* = 47).

	Never (*n* | %)		Rarely (*n* | %)		Sometimes (*n* | %)		Often (*n* | %)			Always (*n* | %)
In the last 6 months, how likely were your feelings of stress, anxiety and/or sadness/depression to impact your self-care in areas of?										
Grooming	14	29.8	36.2	17	13	27.7	3	6.4	0	0
**Dressing**	15	31.9	38.3	18	11	23.4	3	6.4	0	0
**Eating**	6	12.8	19.2	9	23	48.9	8	17.0	1	2.1
**Bathing**	21	44.7	34.0	16	9	19.2	1	2.1	0	0
**Sleeping**	6	12.8	10.6	5	19	40.4	15	31.9	2	4.3
In the last 6 months, how likely were your feelings of stress, anxiety and/or sadness/depression to impact your ability to participate in areas of?										
**Academic work**	1	2.13	9	19.15	22	46.81	14	29.79	1	2.13
**Cleaning**	5	10.64	14	29.79	15	31.91	9	19.15	3	6.38
**Shopping**	8	17.02	13	27.66	14	29.79	10	21.28	0	0
**Cooking**	5	10.64	13	27.66	17	36.17	11	23.40	0	0
**Managing finances**	9	19.15	21	44.68	13	27.66	2	4.26	1	2.13
**Work**	5	10.64	11	23.40	8	17.02	4	8.51	1	2.13
**On/off campus volunteering**	8	17.02	7	14.89	9	19.15	5	10.64	0	0
In the last 6 months, how likely were your feelings of stress, anxiety and/or sadness/depression to impact your participation in the following leisure activities?										
**Getting out of your home**	8	17.0	18	38.3	14	29.79	7	14.9	0	0
**Physical activity**	5	10.6	12	25.5	17	36.17	11	23.4	2	4.3
**Socializing**	4	8.5	9	19.2	24	51.06	9	19.2	1	2.1
**Maintaining relationships**	5	10.6	19	40.4	13	27.66	10	21.3	0	0
**Making new relationships**	3	6.4	13	27.7	16	34.04	11	23.4	3	6.4
**Sexual activities**	9	19.2	9	19.2	13	27.66	4	8.5	2	4.3
**Hobbies/Other**	4	8.5	8	17.0	24	51.06	10	21.3	0	0

### Productivity/work

Participants rated that their feelings would ‘never’ (*n* = 41, 12.46%), ‘rarely’ (*n* = 88, 26.75%), ‘sometimes’ (*n* = 98, 29.79%), ‘often’ (*n* = 55, 16.72%) or ‘always’ (*n* = 6, 1.82%) impact their engagement inproductivity. Mental health was ranked ‘sometimes’ or ‘often’ to impact participants’ engagement in many productivity occupations, especially academic work. Respondents rated that their feelings would ‘sometimes’ (*n* = 22, 46.81%) or ‘often’ (*n* = 14, 29.79%) impact their participation in academic work.

### Leisure/play

Respondents rated that their feelings would ‘never’ (*n* = 38, 12.0%), ‘rarely’ (*n* = 88, 27.8%), ‘sometimes’ (*n* = 121, 38.2%), ‘often’ (*n* = 62, 19.6%) or ‘always’ (*n* = 8, 2.5%) impact their engagement in leisure/play activities. The majority of participants rated that their mental health would ‘sometimes’ or ‘often’ impact their engagement in all leisure/play activities except for maintaining relationships and getting out of the house. Maintaining relationships was ‘rarely’ impacted (*n* = 19, 40.4%) and getting out of the house was rated ‘rarely’ to be impacted (*n* = 18, 38.3%).

### Use of smartphone

Twenty-seven out of 42 students (64.3%) reported that they have used a mental health app on their smartphone. 20 out of 25 participants (80%) engaged with the app weekly to monthly. The most commonly used apps included mindfulness and meditation (*n* = 16, 53.3%), social support (*n* = 4, 13%), relaxation (*n* = 3, 10%), distraction (games, music and social media (*n* = 3, 10%) and mood and thought tracking (*n* = 3, 10%). Common reasons for discontinuing use of these apps included the app being ‘too time consuming’ (*n* = 8, 38%) and cost (*n* = 6, 28.6%).

Forty-one students ranked the features of a smartphone app by importance. The cost of the smartphone app was ranked as most important by 63.4% (*n* = 26) of students, followed by an easy to learn to use app (*n* = 7, 17.1%). The system’s ability to work without manual input from the user was ranked as the least important feature by 39% of participants. However, when asked about their data collection preference, 50% (*n*v28) of participants preferred data being collected automatically. Table 3 provides respondents’ preference rating of possible app outputs and revealed that behavioural cues were most preferred by 42.9% (*n* = 18), followed by data summary (*n*v14, 33.3%), educational content (23.8, *n* = 10), and a list of mental health providers (*n* = 3, 7.1%).

**Table 3.** Possible outputs that could be provided by a smartphone app to support students’ mental health.

	Least preferable (*n* | %)		Not preferable (*n* | %)		Neutral (*n* | %)		Preferable (*n* | %)		Most preferable (*n* | %)	
**List of mental health providers in your area**	1	2.4	2	4.8	15	35.7	21	50.0	3	7.1
**Behavioural change cues (i.e. notifications to walk, stand up, deep breath, call a friend, etc.**	0	0	1	2.4	6	14.3	17	40.5	18	42.9
**Data summary (i.e. sleep log, HR, social media phone usage)**	0	0	1	2.4	5	11.9	22	52.4	14	33.3
**Educational content on mental health (i.e. videos, articles**	1	2.4	1	2.4	13	31.0	17	40.5	10	23.8

## Discussion

This study provides a greater understanding of what features and functions to include and what data to collect, when developing a novel app to support students’ mental health and engagement in activities. The majority of students reported living with someone, indicating they had social support which is known to positively impact mental health.(Berkman et al., 2000) The discrepancy between the number of reported mental health issues and the amount of reported mental health symptoms may suggest that students in this study were unwilling to identify their mental health support needs. Our findings suggest previous studies (American College Health Association, 2019) may be under reporting the actual number of mental health issues among post-secondary studies. Exacerbating matters, initial studies indicate that the COVID-19 pandemic has negatively influenced the mental health of post-secondary students (Cao et al., 2020; Statistics Canada, 2020; Patterson et al., 2021). Post-secondary students often choose not to disclose their mental health issues either because they believe their issue is not serious enough, because they fear repercussions due to the stigma associated with mental health in higher education (Clement et al., 2015) or because of cultural considerations (Yang et al., 2007). Ultimately, this results in reduced help-seeking behaviour in students.(Venville et al., 2014) Since students are currently using their smartphones to support their mental health, an app designed to predict and alert students to mental health changes and provide real-time support has great potential.

This study describes the association between mental health changes and self-care engagement, productivity, and leisure/play domains. Firstly, it demonstrates mental health changes impact students’ engagement in all activities in some occupational domains, but only some activities in other domains. Changes in mental health affect many activities within leisure/play and productivity domains such as academic productivity. Secondly, it demonstrates that in addition to psychological and physiological changes, changes in engagement in daily activities often indicate to students that they are experiencing a change in their mental health. These indicators are congruent with the body of literature regarding global positioning system and Bluetooth data, which states that location entropy, low co-location rates and poor sleep quality are strong indicators of mental health changes.(Abdullah & Choudhury, 2018; Fraccaro et al., 2019) Therefore, developers should design the app to track this information to determine when outputs to support mental health and engagement in activities are needed.

The most preferred output features for a new app included data summary and behavioural cues. Developers can use this study’s preliminary results regarding the relationship between mental health and engagement in activities to guide future research when developing app outputs. A data summary can provide students with a look at changes in their mental health and its relationship to other data, including engagement in activities. These data summary reports can help students better understand these relationships and gradually build activity routines that will positively impact their mental health. Behavioural cue outputs, such as ‘go for a walk’ to support leisure engagement or ‘join a meditation session’ to support healthy sleep, should be designed to encourage engagement in activities that are typically impacted by changes in or have positive impacts on mental health (e.g. leisure/play and productivity occupations, sleep, and eating). The behavioural cue outputs should also encourage students to engage less in activities that are indicators of negative changes in mental health (e.g. increased screen time and use of a user’s mobile phone). By doing this, behavioural cues would encourage occupational balance or engagement in activities across all occupational domains, which have been shown to have favourable effects on health and well-being (Backman, 2004).

Students identified key ‘must-have’ and ‘preferred’ features and functions to include in an app designed to support their mental health. This study indicates that app developers should consider ease of app use and its ability to work in the background to collect data as ‘must have’ features. Ease of app use features will reduce the amount of time required to manually input data. Furthermore, this would be beneficial as there was a higher reported preference for automatic input of data (e.g. biometric), with manual engagement on a weekly-monthly basis rather than daily. Besides being time-consuming, the cost of the app or ‘in-app purchases’ also played a great role in the discontinued use of apps, suggesting a new app should have zero to minimal costs associated with it.

The study had several limitations. Firstly, the convenience sample (e.g. largely female graduate students at one Canadian university) and self-reported outcomes of the study limit its external validity of the findings. The cross-sectional design used to suggest the results in this study represent a point in time and may not exist over time (Levin, 2006). Given that this is a pilot study, further research with larger sample size is warranted. The development and use of a new instrument for student mental health and engagement in activities warrants an evaluation of the instruments’ psychometric properties. The survey was administered during the COVID-19 pandemic, which could act as a confounding variable affecting participants’ mental health and engagement in activities.

## Conclusion

Our study demonstrates the feasibility of post-secondary students use and preferences of important features and functions of smartphone applications that support their mental health and engagement in activities. Our study identified specific content that will maximize functioning related to self-care, productivity and play activities as important determinants of mobile mental health apps among post-secondary students. While developing a new app, it may be beneficial for developers to consider how students are already using their smartphones to support their mental health. In addition to creating unique features and functions for a novel app, developers may also consider the potential usefulness of incorporating features and functions that students already use to support mental health. Future studies should consider using larger sample sizes, and following students over a longer period of time in understanding these determinants through a mental health and functioning perspective and their effectiveness in important engagement in activities. With further research, a highly useful app will be developed to improve mental health interventions and increase post-secondary students’ engagement in meaningful activities that will improve their mental health and well-being.
